# Antifungal Activity of Ag(I) and Zn(II) Complexes of Sulfacetamide Derivatives

**DOI:** 10.1155/MBD.2000.49

**Published:** 2000

**Authors:** Antonio Mastrolorenzo, Claudiu T. Supuran

**Affiliations:** 1 Universitá degli Studi Istituto di Clinica Dermosifilopatica Centro MTS, Via degli Alfani 37 Firenze I-50122 Italy; 2 Universitá degli Studi Dipartamento di Chimica Laboratorio di Chimica Inorganica e Bioinorganica Via Gino Capponi 7 Firenze I-50121 Italy

## Abstract

Reaction of sulfacetamide with arylsulfonyl isocyanates afforded a series of derivatives which were used as ligands (as conjugate bases) for the preparation of metal complexes containing Ag(I) and Zn(II). The newly synthesized complexes, unlike the free ligands, act as effective antifungal agents against *Aspergillus* and *Candida spp.*, some of them showing activities comparable to ketoconazole, with minimum inhibitory concentrations in the range of 0.3 – 0.5 μg/mL. The mechanism of antifungal action of these complexes seems to be not connected with the inhibition of lanosterol-14-α-demethylase, since the levels of sterols assessed in the fungi cultures were equal in the absence or in the presence of the tested compounds. Probably the new complexes act as inhibitors of phosphomannose isomerase, a key enzyme in the biosynthesis of yeast cell walls.


### ANTIFUNGAL ACTIVITY OF Ag(I) AND Zn(II) COMPLEXES OF SULFACETAMIDE DERIVATIVES

Antonio Mastrolorenzo[1]* and Claudiu T. Supuran[2]

[1] Università degli Studi, Istituto di Clinica Dermosifilopatica, Centro MTS, Via degli Alfani 37, I-50122 Firenze, Italy; Fax: +39-055-2758395; E-mail: amas@dada.it
[2] Università degli Studi, Dipartamento di Chimica, Laboratorio di Chimica Inorganica e Bioinorganica, Via Gino Capponi 7, I-50121, Firenze, Italy; E-mail: cts@bio.chim.unifi.it

**Abstract**: Reaction of sulfacetamide with arylsulfonyl isocyanates afforded a series of derivatives which were used as ligands (as conjugate bases) for the preparation of metal complexes containing Ag(I) and Zn(II). The newly synthesized complexes, unlike the free ligands, act as effective antifungal agents against *Aspergillus* and *Candida spp.*, some of them showing activities comparable to ketoconazole, with minimum inhibitory concentrations in the range of 0.3 – 0.5 μg/mL. The mechanism of antifungal action of these complexes seems to be not connected with the inhibition of lanosterol-14-α-demethylase, since the levels of sterols assessed in the fungi cultures were equal in the absence or in the presence of the tested compounds. Probably the new complexes act as inhibitors of phosphomannose isomerase, a key enzyme in the biosynthesis of yeast cell walls.

### Introduction

Several important classes of antifungal compounds are presently available,[1-5] such as the azoles inhibiting lanosterol-14-α-demethylase,[1-5] sterol-$\Delta^{14}$-reductase,[1] or $\Delta^7$-$\Delta^8$ isomerase [2] as well as the inhibitors of the zinc enzymes phosphomannose isomerase,[6a,b] or topoisomerase.[6c] All these enzymes are involved in the biosynthesis of fungi/yeast cell walls, and their inhibition leads to impaired function of the membrane and as a consequence death of the pathogenic species. Recently, some metal complexes such as silver sulfadiazine were shown to possess effective antifungal properties against the pathogenic yeast *Candida albicans*.[6,7,8] The mechanism of action of this complex seems to be connected with the inhibition of phosphomannose isomerase, a key enzyme in the biosynthesis of yeast cell walls. [6,7,8]

Since resistance to the different antifungal agents constantly emerges,[2-5,9-11] it is important to investigate new types of compounds, able to prevent this serious medical problem. Metal complexes, containing mainly Ag(I) and Zn(II) seem to be a valuable such alternative.[7,8,12-15] Indeed, silver sulfadiazine **1** is extensively used clinically for the prophylaxis and treatment of bacterial and fungal burn infections, alone or in combination with mafenide acetate **2**, as well as cerium(IV) nitrate.[8,12-15]

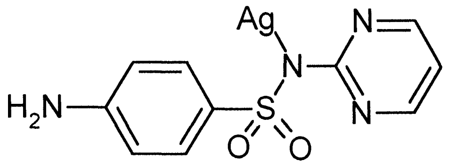

**1**

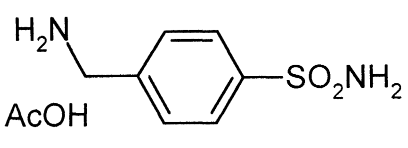

**2**

Considering compound **1** as lead molecule, we prepared some new derivatives of sulfacetamide **3**, another widely used clinical agent,[16] by its reaction with arylsulfonyl isocyanates of type **4**.[17] The conjugate bases of the new derivatives **5-9** were then used as ligands for the preparation of the Ag(I) and Zn(II) complexes **10-19**. The new compounds and their complexes were assayed for their antifungal properties against two *Aspergillus* and one *Candida albicans* strains.

### Materials and Methods

Elemental analyses were done by combustion with a Carlo Erba Instrument or gravimetrically for the metal ions. NMR spectra have been recorded at 200 MHz, with a Varian Gemmini 200 spectrophotometer; chemical shifts are reported as δ values, relative to Me4Si as external standard, in solvents specified in each case. IR spectra were recorded with a Perkin-Elmer spectrophotometer using KBr pellets as reference. Conductimetric measurements were done at room temperature on a Radelkis KFT conductimeter.

Sulfacetamide, sulfadiazine, mafenide hydrochloride, arylsulfonyl isocyanates, metal salts and solvents were commercially available (from Sigma, Aldrich or Janssen) and were used without further purification.

*General procedure for preparation of arylsulfonylureido sulfacetamides* **5-9**

An amount of 2.15 g (10 mM) of sulfacetamide was suspended in 100 mL of highly anhydrous (kept on molecular sieves) acetonitrile and magnetically stirred at 4°C for 10 min. The stoichiometric amount of arylsulfonyl isocyanate **4** (eventually dissolved in the same solvent for the solid compounds, or in pure state for the liquid ones) was then added dropwise, maintaining the temperature under 10 °C. The reaction mixture was stirred at room temperature for 2-4 h (tlc control), then the solvent was evaporated in vacuo and the residue crystallized from ethanol-water. Yields were practically quantitative.

*4-(Benzenesulfonylamidocarbonyl)-sulfacetamide*, **5**: colorless crystals, mp 295-6 °C (dec.). IR(KBr), $cm^{-1}$: 1128 and 1176 ($SO_2^{sym}$), 1290 (amide III), 1355 and 1382 ($SO_2^{as}$), 1520 and 1540 (amide II); 1680 (amide I), 3065 and 3190 (NH); $^1$H-NMR (DMSO-$d_6$), δ, ppm: 2.75 (s, 3H, AcN); 7.05 – 7.65 (m, 5H, Ph); 7.46 –8.10 (m, AA'BB', $J_{AB}$ = 7.6 Hz, 4H, $C_6H_4SO_2$); 7.94 (s, 1H, $SO_2NH$); 8.40 (s, 1H, CON*H*-); 10.63 (br s, 1H, $SO_2NH$); Analysis, found: C, 45.50; H, 3.92; N, 10.43 %; $C_{15}H_{15}N_3O_6S_2$ requires: C, 45.33; H, 3.80; N, 10.54 %.

*4-(4-Fluorobenzenesulfonylamidocarbonyl)-sulfacetamide*, **6**: colorless crystals, mp 250-2 °C (dec.). IR(KBr), $cm^{-1}$: 1130 and 1175 ($SO_2^{sym}$), 1291 (amide III), 1355 and 1380 ($SO_2^{as}$), 1520 and 1540 (amide II); 1680 (amide I), 3065 and 3190 (NH); $^1$H-NMR (DMSO-$d_6$), δ, ppm: 2.77 (s, 3H, AcN); 7.02 – 7.50 (m, AA'BB', $J_{AB}$ = 7.1 Hz, 4H, *p*-F-phenylene); 7.55 –8.14 (m, AA'BB', $J_{AB}$ = 7.6 Hz, 4H, $C_6H_4SO_2$); 7.98 (s, 1H, $SO_2NH$); 8.44 (s, 1H, CON*H*-); 10.60 (br s, 1H, $SO_2N$*H*c); Analysis, found: C, 43.24; H, 3.25; N, 10.09 %; $C_{15}H_{14}FN_3O_6S_2$ requires: C, 43.37; H, 3.40; N, 10.12 %.

*4-(4-Chlorobenzenesulfonylamidocarbonyl)-sulfacetamide*, **7**: colorless crystals, mp 269-70 °C (dec.). IR(KBr), $cm^{-1}$: 1135 and 1174 ($SO_2^{sym}$), 1290 (amide III), 1354 and 1378 ($SO_2^{as}$), 1520 and 1540 (amide II); 1680 (amide I), 3065 and 3190 (NH); $^1$H-NMR (DMSO-$d_6$), δ, ppm: 2.75 (s, 3H, AcN); 7.02 – 7.53 (m, AA'BB', $J_{AB}$ = 7.2 Hz, 4H, *p*-Cl-phenylene); 7.57 –8.11 (m, AA'BB', $J_{AB}$ = 7.6 Hz, 4H, $C_6H_4SO_2$); 7.96 (s, 1H, $SO_2NH$); 8.41 (s, 1H, CON*H*); 10.58 (br s, 1H, $SO_2NH$); Analysis, found: C, 41.54; H, 3.47; N, 9.60 %; $C_{15}H_{14}ClN_3O_6S_2$ requires: C, 41.72; H, 3.27; N, 9.73 %.

*4-(4-Tosylamidocarbonyl)-sulfacetamide*, **8**: colorless crystals, mp 280-2 °C (dec.). IR(KBr), $cm^{-1}$: 1125 and 1176 ($SO_2^{sym}$), 1290 (amide III), 1355 and 1380 ($SO_2^{as}$), 1520 and 1540 (amide II); 1680 (amide I), 3065 and 3190 (NH); $^1$H-NMR (DMSO-$d_6$), δ, ppm: 2.50 (s, 3H, *p*-tosyl); 2.75 (s, 3H, AcNH); 7.05 – 7.59 (m, AA'BB', $J_{AB}$ = 7.2 Hz, 4H, phenylene from tosyl); 7.46 –8.08 (m, AA'BB', $J_{AB}$ = 7.6 Hz, 4H, $C_6H_4SO_2$); 7.91 (s, 1H, $SO_2NH$); 8.42 (s, 1H, CON*H*); 10.69 (br s, 1H, $SO_2NH$); Analysis, found: C, 46.50; H, 3.98; N, 10.25 %; $C_{16}H_{17}N_3O_6S_2$ requires: C, 46.71; H, 4.16; N, 10.21 %.

*4-(2-Tosylamidocarbonyl)-sulfacetamide*, **9**: colorless crystals, mp 213-4 °C. IR(KBr), $cm^{-1}$: 1134 and 1173 ($SO_2^{sym}$), 1290 (amide III), 1341 and 1380 ($SO_2^{as}$), 1520 and 1540 (amide II); 1680 (amide I), 3065 and 3190 (NH); $^1$H-NMR (DMSO-$d_6$), δ, ppm: 2.59 (s, 3H, *o*-tosyl); 2.75 (s, 3H, AcN); 7.05 – 7.76 (m, 4H, phenylene from *o*-tosyl); 7.49 –8.06 (m, AA'BB', $J_{AB}$ = 7.6 Hz, 4H, ArH from $C_6H_4SO_2$); 7.95 (s, 1H, $SO_2NH$); 8.50 (s, 1H, CON*H*); 10.62 (br s, 1H, $SO_2NH$); Analysis, found: C, 46.87; H, 4.22; N, 10.13 %; $C_{16}H_{17}N_3O_6S_2$ requires: C, 46.71; H, 4.16; N, 10.21 %.

*General procedure for the preparation of complexes* **10-19**

An amount of 6 mmol of sodium salt of sulfonamides **5-9** was prepared by reacting the corresponding sulfonamide with the required amount of an alcoholic 1N NaOH solution, in ethanol as solvent. To this solution was added the aqueous metal salt solution (Zn(II), Ag(I) nitrates), working in molar ratios sulfacetamide derivative: $M^{n+}$ of 2:1 for the zinc compounds, and 1:1 for the silver derivatives, respectively. The aqueous-alcoholic reaction mixture was heated on a steam bath for one hour, adjusting the pH at 7 if necessary, and after being cooled at 0 °C the precipitated complexes were filtered and thoroughly washed with alcohol-water 1:1 (v/v) and air dried. Yields were in the range of 85-90 %. The obtained white or yellowish powders of compounds **10-19** melted with decomposition at temperatures higher than 350 °C, and were poorly soluble in water and alcohol, but had good solubilities in DMSO, DMF as well as mixtures of DMSO-water, DMF-water.

*Assay of fungistatic activity of compounds* **1-19**

The fungistatic activity was determined by a modification of the growth method recently reported,[18-21] utilizing two *Aspergillus* and one *Candida spp.* Minimum inhibitory concentrations (MICs) have been determined by the agar dilution method with Iso-Sensitest agar as described by Kinsman et al.[22] The fungi/moulds were cultivated in agar plates at 37°C for 5 days, in the nutrient broth (NB, Diagnostic Pasteur), in the absence and in the presence of 100 - 0.01 μg/mL of compounds **1-19**. Stock solutions of inhibitors were obtained in DMSO (100 mg/mL) and dilutions up to 0.01 μg/mL were done with distilled deionized water. The minimum concentration at which no growth was observed was taken as MIC value (μg/mL), and represents the mean of at least two determinations.

*Assay of sterols present in the fungi cultures*

A reverse-phase HPLC method adapted from the literature,[23] has been used to determine the amount of sterols (ergosterol and lanosterol) present in the fungi cultures. The fungi have been cultivated as mentioned above for 5 days, with or without inhibitors added in the nutrient broth. Culture media were suspended in a small volume of MOPS buffer (pH 7.4) and the cells centrifuged at 20000g for 30 min. Cells were weighed (wet paste) and broken by sonication. Sterols present in the homogenate were then extracted in chloroform, the solvent has been evaporated to a small volume and the extracts applied on a μ-Bondapak-C18 column, with acetonitrile as eluting solvent. Authentic ergosterol and lanosterol (from Sigma) were used as standards. The flow rate was of 3 mL/min. The retention times were: 8.87 min for ergosterol; and 7.62 min for lanosterol, respectively. Blank assay were done for cultures which were not treated with inhibitors in order to assess the normal levels of sterols present. The amount of ergosterol present in the same amount of wet cells from the culture grown in the absence of inhibitor was taken as 100%.[21-24]

**Results and Discussion**

Reaction of sulfacetamide **3** with arylsulfonyl isocyanates **4**, afforded the ureido derivatives **5-9**, by the procedure already reported previously by this group.[17] The new compounds obtained as outlined in Scheme 1, were characterized by spectroscopic and analytic data that confirmed their structures.

Metal complexes **6-11** containing the conjugate base of sulfonamides **5-9** (LH) and Ag(I) or Zn(II) ions, of types **10-19**, were also obtained (Scheme 1), and their elemental analysis data are shown in Table I.

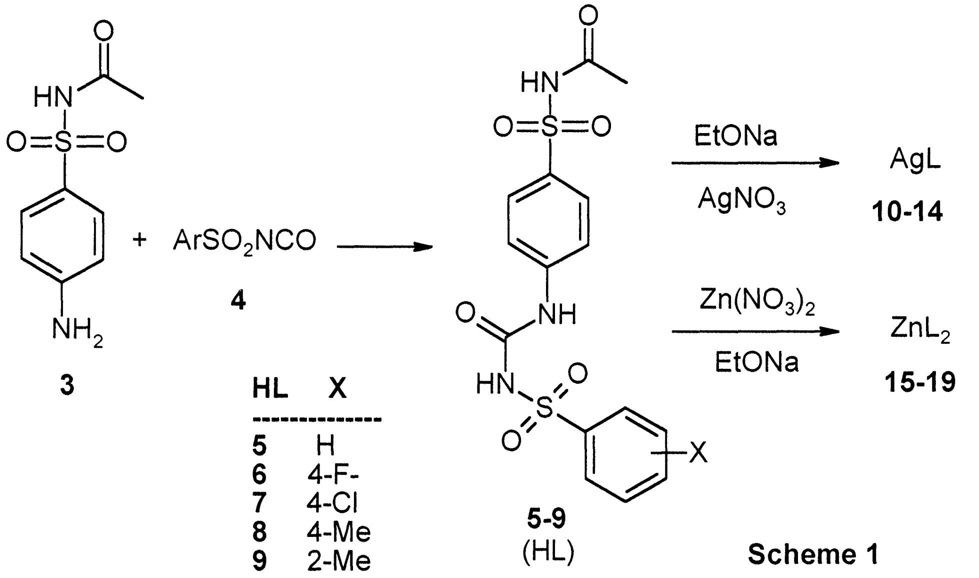

**Scheme 1**

The new complexes have been characterized by spectroscopic, conductimetric and thermogravimetric measurements (Table II). By comparing the IR spectra of the complexes and the free ligand, the following differences were evidenced: (i) the shift of the two sulfonamido vibrations (both the symmetric as well as the asymmetric one) belonging to the $SO_2NHAc$ moiety (at 1173-1176 $cm^{-1}$, and 1378-1382 $cm^{-1}$) towards lower wavenumbers in the spectra of the complexes, as compared to the spectra of the corresponding ligand (Table II), as already documented previously for similar complexes.[25-29] One should note that only one pair of such vibrations underwent the above-mentioned shift, presumably those of the $SO_2NHAc$ moiety, whereas the second sulfonamide ($X$-$C_6H_4SO_2NHCONH$) moiety appeared at the same wavenumbers both in ligands as well as in their metal complexes (data not shown). This is a direct indication that the deprotonated $SO_2NHAc$ moiety of the ligand interacts with the metal ions in the newly prepared coordination compounds; (ii) the acetamide stretching vibration in the spectra of the prepared complexes are shifted with 15-20 $cm^{-1}$ towards lower wavenumbers, as compared to the same vibration in the spectrum of sulfonamides **5-9** indicating again that this moiety is probably in the vicinity of the metal ions (data not shown); (iii) the ureido vibration in the spectra of complexes **10-19**, assigned as the intense band at 1680 $cm^{-1}$ appear at the same wavelength as that of the corresponding free ligands (Table II), suggesting that these moieties do not participate in the coordination of the metal ions.

Table I: Complexes **10-19**, containing the conjugate base of sulfonamides **5-9** (LH) as ligand and their elemental analysis data.

| No. | Complex | Ligand (LH) | Analysis (calculated/found) %M[a] | %C[b] | %H[b] | %N[b] |
|---|---|---|---|---|---|---|
| **10** | [AgL] | **5** | 21.39/21.50 | 35.73/35.61 | 2.80/2.71 | 8.33/8.30 |
| **11** | [AgL] | **6** | 20.65/20.32 | 34.50/34.29 | 2.51/2.36 | 8.05/7.84 |
| **12** | [AgL] | **7** | 20.02/20.30 | 33.44/33.25 | 2.43/2.21 | 7.80/7.51 |
| **13** | [AgL] | **8** | 20.81/20.64 | 37.08/36.92 | 3.11/3.19 | 8.11/8.03 |
| **14** | [AgL] | **9** | 20.81/20.49 | 37.08/37.15 | 3.11/3.45 | 8.11/7.87 |
| **15** | [$ZnL_2$] | **5** | 7.62/7.45 | 41.99/42.26 | 3.28/3.07 | 9.79/9.63 |
| **16** | [$ZnL_2$] | **6** | 5.06/5.35 | 41.85/41.71 | 3.20/3.37 | 9.76/9.69 |
| **17** | [$ZnL_2$] | **7** | 4.94/4.72 | 40.81/40.96 | 3.12/3.38 | 9.52/9.43 |
| **18** | [$ZnL_2$] | **8** | 5.09/5.40 | 43.98/43.62 | 3.69/3.54 | 9.82/9.67 |
| **19** | [$ZnL_2$] | **9** | 5.09/4.96 | 43.98/43.95 | 3.69/3.77 | 9.82/9.71 |

[a]By gravimetry; [b]By combustion.

One may reach the same conclusion by studying the $^{1}$H-NMR spectra of the Ag(I) and Zn(II) complexes, as compared to the corresponding spectra of the ligands. Thus, the only difference between the two types of spectra concerns the signal of the methyl group of the $AcNHSO_2$ moiety, which in the complexes appears at 2.55 – 2.68 ppm, whereas in the ligands at 2.75-2.77 ppm (Table II). Similar behaviors were also evidenced previously for other sulfonamide metal complexes, by our group, [25-29] and probably indicate that this methyl moiety is in the vicinity of the zinc/silver ions in the prepared complexes. Conductometric data (Table II) also indicate that the new complexes **10-19** are non-electrolytes, being undissociated in DMF (or DMSO) as solvent.

Biological activity data with the new derivatives **1-19** and the standard azole antifungal agent ketoconazole **20** are shown in Table III.

Table II: Spectroscopic, thermogravimetric and conductimetric data for compounds **4-11**.

| **Comp.** | **IR Spectra**[a], $cm^{-1}$ $\nu (SO_2)^s$ | $\nu (SO_2)^{as}$ | $\nu$ (C=O)[d] | **$^{1}$H-NMR spectra**[b] $\delta$, ppm (Me of $AcNHSO_2$) | **Conductometry**[c] $\Lambda_M$ ($\Omega^{-1}$ x $cm^2$ x $mol^{-1}$) |
|---|---|---|---|---|---|
| **10** | 1161 | 1368 | 1680 | 2.64 | 3 |
| **11** | 1158 | 1366 | 1680 | 2.63 | 6 |
| **12** | 1157 | 1365 | 1680 | 2.68 | 6 |
| **13** | 1160 | 1365 | 1680 | 2.61 | 5 |
| **14** | 1159 | 1365 | 1680 | 2.62 | 5 |
| **15** | 1155 | 1360 | 1680 | 2.60 | 4 |
| **16** | 1154 | 1360 | 1680 | 2.57 | 8 |
| **17** | 1155 | 1357 | 1680 | 2.55 | 3 |
| **18** | 1155 | 1356 | 1680 | 2.58 | 4 |
| **19** | 1154 | 1361 | 1680 | 2.57 | 6 |

[a] In KBr; [b]In DMSO, at 25 °C; [c] 1 mM solution, in DMF, at 25°C; [d] Probably the ureido NHCONH vibration.

From data of Table III, one should note that the new complexes **10-19** reported here represent a new class of antifungals with MIC-s (minor inhibitory concentration) in the micromolar range, which might induce strong *in vivo* antifungal effects. Furthermore, the ligands from which the complexes were prepared, as well as the related sulfonamide **2**, are devoid of such antifungal properties, against the three strains investigated here. The most active derivative were the Ag(I) complexes **10-14**, followed by the Zn(II) ones containing the same type of ligand. From this point of view, the halogeno-substituted ligands led to more active antifungal complexes as compared to the phenyl- and tosyl-ureido derivatives. *Candida* was most susceptible to inhibition, followed by *A. flavus*, whereas *A. niger* was the most resistant to this type of antifungals. In this respect, the complex derivative parallel the biological activity of ketoconazole, although they are less active. One should anyhow note that some of our new complexes are more active antifungals than silver sulfadiazine **1**, a clinically widely used derivative (Table III).

Ketoconazole **20** is known to act as an inhibitor of lanosterol 14-α-demethylase (CYP51A1), a microsomal cytochrome P-450 dependent enzyme system belonging to a gene superfamily involved in sterol biosynthesis in fungi, plants and animals.[24] CYP51A1 has been shown to catalyze the conversion of lanosterol to the 14-desmethylated derivative, ergosterol, through a complicated oxidative sequence. Its inhibition in fungi causes the depletion of ergosterol and accumulation of 14-methylsterols in the membrane of the cells, disturbing thus membrane function and causing the death of these organisms.[24]

Table III: Antifungal activity of compounds **1-19** against several organisms.

| Compound | MIC (μg/mL) | | |
|---|---|---|---|
| | *A. flavus* C1150 | *A. niger* C418 | *Candida albicans* C316 |
| **1** | 20 | 23 | 8 |
| **2** | >100 | >100 | >100 |
| **3** | >100 | >100 | >100 |
| **5** | >100 | >100 | >100 |
| **6** | >100 | >100 | >100 |
| **7** | >100 | >100 | >100 |
| **8** | 95 | >100 | 84 |
| **9** | 88 | >100 | 87 |
| **10** | 12 | 13 | 2.5 |
| **11** | 9 | 10 | 1.8 |
| **12** | 3 | 5 | 1.9 |
| **13** | 5 | 7 | 2.4 |
| **14** | 9 | 10 | 2.8 |
| **15** | 22 | 29 | 6 |
| **16** | 18 | 24 | 5 |
| **17** | 13 | 25 | 5 |
| **18** | 21 | 27 | 9 |
| **19** | 18 | 19 | 11 |
| **Ketoconazole 20** | 1.2 | 1.8 | 0.06 |

Thus, in order to investigate whether the complexes reported here act as ergosterol biosynthesis inhibitors, similarly to the azole antifungals, the amounts of ergosterol present in *C. albicans* cultures after treatment with different concentrations of the new the inhibitor **11** and ketoconazole **20**, a potent CYP51A1 inhibitor,[20] have been determined by means of a HPLC method (Table IV).[23]

Table IV: Levels of ergosterol in *C. albicans* cultures after treatment with different concentrations of the azole CYP51A1 inhibitor ketoconazole **20** and the silver complex **11**.

| Inhibitor | Concentration (μg/mL) | % Ergosterol* |
|---|---|---|
| Ketoconazole | 0.01 | 89 ± 5 |
| Ketoconazole | 0.02 | 66 ± 7 |
| Ketoconazole | 0.05 | 8 ± 4 |
| **11** | 0.25 | 99 ± 1 |
| **11** | 0.50 | 98 ± 2 |
| **11** | 1.00 | 98 ± 2 |
| **11** | 1.50 | 99 ± 2 |

*Mean ± standard deviation (n = 3); The amount of ergosterol present in the same amount of wet cells from the culture grown in the absence of inhibitor is taken as 100 %.

The data of Table IV show that, in contrast to ketoconazole, the silver complex investigated here, **11**, does not act as inhibitor of CYP51A1, possessing thus a different mechanism of antifungal action. Probably, as many other Ag(I) derivatives, the new antifungals might exert their effects through poisoning some components of the respiratory enzymes in the fungal/microbial electron transport system, or even through interaction with the DNA of the pathogenic species.[30,31]

In conclusion we report here the preparation and antifungal activity of some Ag(I) and Zn(II) complexes of sulfacetamide derivatives, possessing interesting biological activity against several *Aspergillus* and *Candida* strains.

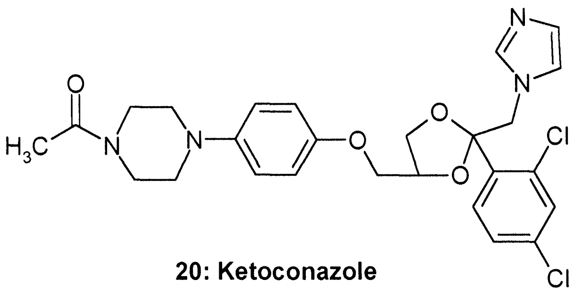

**20: Ketoconazole**

### References

1. Berg, D., Plempel, M. (1994) Fungal Enzyme Targets. In "*Design of Enzyme Inhibitors as Drugs*", 2nd Edition, Sandler, M., Smith, H.J. Eds., Oxford Univ. Press, pp. 364-377.
2. Georgopapadakou, N.H. (1998) *Curr. Opin. Microbiol.* **1**, 547-557.
3. Vanden Bossche, H., Marichal, P., Odds, F.C. (1994) *Trends Microbiol.* **2**, 393-400.
4. Vanden Bossche, H., Koymans, L., Moereels, H. (1995) *Pharmacol. Ther.* **67**, 79-100.
5. Asai, K., Tsuchimori, N., Okonogi, K., Perfect, J.R., Gotoh, O., Yoshida, Y. (1999) *Antimicrob. Agents Chemother.* **43**, 1163-1169.
6. a) Wells, T.N.C., Scully, P., Paravicini, G., Proudfoot, A.E.I., Payton, M.A. (1995) *Biochemistry* **34**, 7896-7903; b) Cleasby, A., Wonacott, A., Skarzynski, T., Hubbard, R.E., Davies, G.J., Proudfoot, A.E.I., Bernard, A E., Payton, M.A., Wells, T.N. (1996) *Nature Struct. Biol.* **3**, 470-479; c) Fostel, J.M., Montgomery, D.A., Shen, L.L. (1992) *Antimicrob. Agents Chemother.* **36**, 2131-2138.
7. Louie, A.Y., Meade, T.J. (1999) *Chem. Rev.* **99**, 2711-2734.
8. Wright, J.B., Lam, K., Hansen, D., Burrell, R.E. (1999) *Am. J. Infect. Control* **27**, 344-350.
9. Joseph-Thorne, T., Hollomon, D., Loeffler, R.S.T., Kelly, S.L. (1995) *FEBS Lett.* **374**, 174-178.
10. Marichal, P., Vanden Bossche, H. (1995) *Acta Biochim. Pol.* **42**, 509-516.
11. Joseph-Thorne, T., Hollomon, D. (1997) *FEMS Microbiol. Lett.* **149**, 141-149.
12. Singer, A.J., Berrutti, L., McClain, S.A. (1999) *Wound Repair Regen.* **7**, 356-361.
13. Marone, P., Monzillo, V., Perversi, L., Carretto, E. (1998) *J. Chemother.* **10**, 17-21.
14. Fox, C.L., Rao, T.N., Azmeth, R., Gandhi, S.S., Modak, S. (1990) *J. Burn Care Rehabil.* **11**, 112-117.
15. George, N., Faogali, J., Muller, M. (1997) *Burns* **23**, 493-495.
16. a) Northey, E.H. (1948) "The Sulfonamides and Allied Compounds", Reinhold, New York, pp. 1-267; b) Mandell, G.L., Sande, M.A. (1990) "Antimicrobial Agents". In *The Pharmacological Basis of Therapeutics*, 8th Edition, Gilman, A.G., Rall, T.W., Nies, A.S., Taylor P., Eds., Pergamon Press, New York, pp. 1047-1064.
17. Scozzafava, A., Supuran, C.T. (1999) *J. Enzyme Inhib.* **14**, 343-363.
18. Barboiu, M., Guran, C., Jitaru, I., Cimpoesu, M., Supuran, C.T. (1996) *Metal Based Drugs*, **3**, 233-240.
19. a) Supuran, C.T., Scozzafava, A., Briganti, F., Loloiu, G., Maior, O. (1998) *Eur. J. Med. Chem.* **33**, 821-830; b) Supuran, C.T., Scozzafava, A., Briganti, F., Loloiu, G., Maior, O. (1998) *J. Enzyme Inhib.*, **13**, 291-310 ; c) Briganti, F., Scozzafava, A., Supuran, C.T. (1997) *Eur. J. Med. Chem.*, **32**, 901-910.
20. Vanden Bossche, H. (1986) *Drug Dev. Res.* **8**, 287-298.
21. Barboiu, M., Cimpoesu, M., Guran, C., Supuran, C.T. (1996) *Metal Based Drugs* **3**, 227-232.
22. Kinsman, O.S., Livermore, D.G., Smith, C. (1993) *Antimicrob. Agents Chemother.* **37**, 1242-1246.
23. a) Hansbury, E., Scallen, T.J. (1978) *J. Lipid Res.* **19**, 742-746; b) Ruan, B., Gerst, N., Emmons, G.T., Shey, J., Schroepfer, G.J. (1997) *J. Lipid Res.* **38**, 2615-2626.
24. a) Aoyama, Y., Yoshida, Y., Sonoda, Y., Sato, Y. (1991) *Biochim. Biophys. Acta* **1081**, 262-266; b) Aoyama, Y., Yoshida, Y., Sonoda, Y., Sato, Y. (1992) *Biochim. Biophys. Acta* **1122**, 251-255.
25 Supuran, C.T., Scozzafava, A. (1997) *J. Enzyme Inhib.*, **12**, 37-51.
26 a) Mincione, G., Scozzafava, A., Supuran, C.T. (1997) *Metal Based Drugs*, **4**, 27-34; b) Scozzafava, A., Supuran, C.T. (1997) *Metal Based Drugs*, **4**, 19-26.
27 Supuran, C.T., Ilies, M.A., Scozzafava, A. (1998) *Eur. J. Med. Chem.*, **33**, 571-582.
28 Supuran, C.T., Mincione, F., Scozzafava, A., Briganti, F., Mincione, G., Ilies, M.A., (1998) *Eur. J. Med. Chem.*, **33**, 247-254.
29 Supuran, C.T., Scozzafava, A., Saramet, I., Banciu, M.D. (1998) *J. Enzyme Inhib.*, **13**, 177-194.
30. Hamilton-Miller, J.M., Shah, S.; Smith, C. (1993) *Chemotherapy* **39**, 405-409.
31. Modak, S.M., Fox, C.R. (1973) *Biochem. Pharmacol.* **22**, 2391-2404.

**Received: December 3, 1999 - Accepted: January 27, 2000 -**
**Received in revised camera-ready format: February 1, 2000**

